# Over-expression of *KdSOC1* gene affected plantlet morphogenesis in *Kalanchoe daigremontiana*

**DOI:** 10.1038/s41598-017-04387-0

**Published:** 2017-07-17

**Authors:** Chen Zhu, Li Wang, Jinhua Chen, Chenglan Liu, Huiming Zeng, Huafang Wang

**Affiliations:** 1 National Engineering Laboratory for Tree Breeding, College of Biological Sciences and Technology, Beijing Forestry University, Beijing, China; 2 Sivilculture Forestry department, College of Forestry, Beijing Forestry University, Beijing, China; 3 Turfgrass Management department, College of Forestry, Beijing forestry university, Beijing, China

## Abstract

*Kalanchoe daigremontiana* reproduces asexually by producing plantlets along the leaf margin. The aim of this study was to identify the function of the *SUPPRESSOR OF OVEREXPRESSION OF CONSTANS 1* gene in *Kalanchoe daigremontiana* (*KdSOC1*) during plantlet morphogenesis. In this study, *KdSOC1* gene expression was detected at stem cell niche during *in vitro* somatic embryogenesis and plantlet morphogenesis. Disrupting endogenous auxin transportation suppressed the *KdSOC1* gene response. Knockdown of the *KdSOC1* gene caused a defect in cotyledon formation during the early heart stage of somatic embryogenesis. Over-expression (OE) of the *KdSOC1* gene resulted in asymmetric plantlet distribution, a reduced number of plantlets, thicker leaves, and thicker vascular fibers. Higher *KdPIN1* gene expression and auxin content were found in OE plant compared to those of wild-type plant leaves, which indicated possible *KdSOC1* gene role in affecting auxin distribution and accumulation. *KdSOC1* gene OE in *DR5-GUS Arabidopsis* reporting lines resulted in an abnormal auxin response pattern during different stages of somatic embryogenesis. In summary, the *KdSOC1* gene OE might alter auxin distribution and accumulation along leaf margin to initiate plantlet formation and distribution, which is crucial for plasticity during plantlet formation under various environmental conditions.

## Introduction

Since auxin was discovered almost 70 years ago^1^, auxin- and cytokinin-mediated plant growth, including embryogenesis, organ initiation, phyllotaxy, and formation of leaf shape have been studied intensively^2^. An excellent review regarding auxin and cytokinin as a Yin-Yang model (traditional Chinese model in which Yin and Yang balance everything) treats auxin and cytokinin as key regulators of different plant developmental events^3^.

The stem cell niche and formation of leaf shape are excellent examples of elucidating how these two hormone signaling pathways and their downstream gene networks regulate changes during multiple phases of plant growth^4, 5^. Plants harbor two stem cell niches, such as the shoot apical meristem (SAM) and root apical meristem (RAM), which are well-organized structures that regulate above- and below-ground development^6^. The way in which stem cell fate is regulated in the SAM and RAM by different environmental cues, including photoperiod, temperature fluctuations, and environmental stress is the key mechanism that initiates formation of different organs^7, 8^. Many master genes have been identified in past decades, including those regulating stem cell identity maintenance in the SAM (*WUSCHEL* [*WUS*], *SHOOTMERISTEMLESS* [*STM*], and *ZWILLE/PINHEAD/AGO10*) and RAM (*WUSCHEL HOMEOBOX*, *SHORTROOT*, and *SCARECROW*) for initiating lateral organ formation in both stem cell niches (*PINFORMED* [*PIN1*], *CUP-SHAPED COTYLEDON* [*CUC*], and *PLETHORA*). The auxin and cytokinin signaling pathways and downstream networks crosstalk through positive and negative loops that regulate stem cell fate, the SAM and RAM structural patterns, and initiate organogenesis sequences^9^. “Go with the response” describes how auxin metabolism and transport control a developmental event, particularly during morphogenesis of leaf shape^10^. The leaf shape formation process is initiated in the SAM and shows how polar transportation of auxin is mediated by the *PIN1* gene in association with the *CUC2* and *ASYMMETRIC LEAVES 1* (*AS1*) genes, resulting in the oval shape of mature leaves in *Arabidopsis* ^11, 12^. Most studies have focused on discovering the molecular clues to further explain the details of the interaction between auxin and cytokinin.

*Kalanchoe daigremontiana* reproduces asexually to produce plantlets and is regarded as a model plant^13^ in crassulacean acid metabolism research. *Kalanchoe daigremontiana* plantlet morphogenesis is the result of somatic embryogenesis and organogenesis. Knockdown of the *SHOOTMERISTEMLESS* gene in *Kalanchoe daigremontiana* (*KdSTM*) abolishes the formation of plantlets by erasing the pedestal site (the central stem cell niche zone) between leaf blades^14^. An evolutionary mutation in the *LEAFY COTYLEDON 1* (*LEC1*) gene results in aborted seed maturation during zygotic embryogenesis, which is associated with plantlet propagation^15^. Closely following plantlet morphogenesis revealed similar traits, such as hormone or related gene expression, during the well-elucidated zygote embryogenesis of *Arabidopsis*, suggesting that auxin- and cytokinin-mediated growth could also be key during plantlet morphogenesis^16^.

We reported previously that the *KdSOC1* gene, which is a member of the MINICHROMOSOME MAINTENANCE 1, AGAMOUS, DEFICIENS, SERUM RESPONSE FACTOR (MADS) box transcription factor family, is highly expressed during plantlet formation under a long day photoperiod^17^. After years of research on *AtSOC1* gene function in *Arabidopsis*, the *AtSOC1* network of genes controlling flowering was elucidated^18, 19^. However, other studies have reported that *SOC1* affects the duration of dormancy rather than stimulates flowering in kiwifruit^20^. *Kalanchoe daigremontiana* depends on the formation of plantlets for propagation, suggesting that the *KdSOC1* gene might play a novel function during plantlet morphogenesis. In addition, determining whether auxin signaling controls *KdSOC1* expression during plantlet morphogenesis would deepen our understanding of this issue.

In this study, the expression patterns of the *KdSOC1* gene during *in vitro* and *in viv*o somatic embryogenesis and organogenesis were analyzed. The phenotypes and physiological properties after over-expression (OE) and knockdown (RNAi) of the *KdSOC1* gene in *Kalanchoe daigremontiana* transgenic lines were analyzed, including hormone content, relative gene expression profiles, and physiological parameters. In addition, the auxin response pattern and the potential role of the *KdSOC1* gene in auxin transport during somatic embryogenesis were also monitored by the *DR5* reporter. The goal was to identify *KdSOC1* gene function during plantlet morphogenesis.

## Results

### *KdSOC1* gene expression patterns during tobacco callus and shoot induction and plantlet formation in *Kalanchoe daigremontiana*

The activation of the *KdSOC1* gene during plantlet morphogenesis in *Kalanchoe daigremontiana* raises an interesting question as to why a gene that stimulates flowering would be involved in asexual reproduction. We speculated that the *KdSOC1* gene might regulate plantlet morphogenesis through leaf margin somatic embryogenesis. Therefore, we checked the spatial expression of the *KdSOC1* gene by deploying *GUS* gene expression under its own promoter during induction of the tobacco callus (*in vitro* somatic embryogenesis during induction of the callus is similar to plantlet morphogenesis in *Kalanchoe daigremontiana*) and plantlet morphogenesis in *Kalanchoe daigremontiana*.

**Figure 1:**
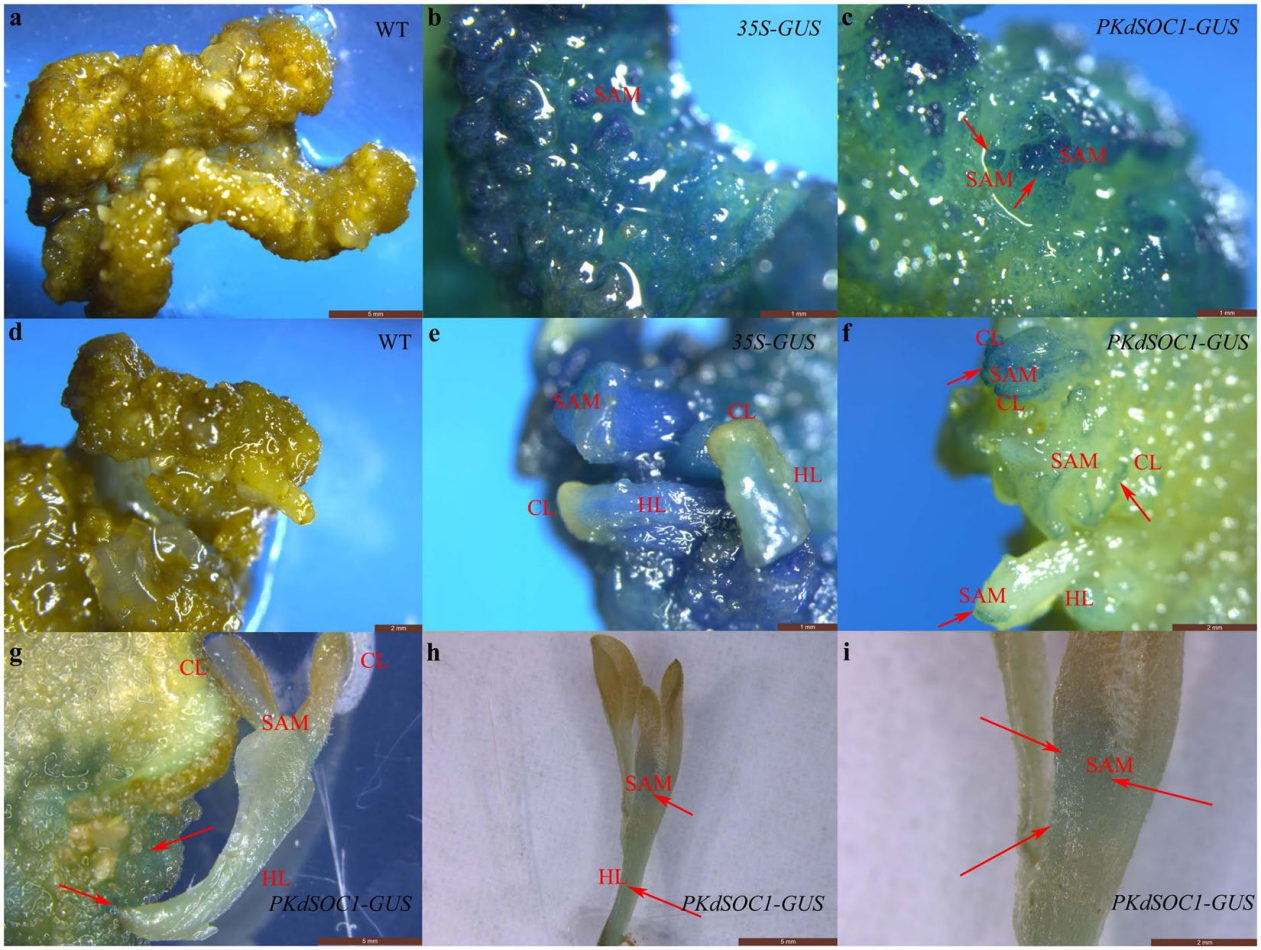
*KdSOC1* gene expression profiles during somatic embryogenesis in tobacco callus induction by GUS staining. (**a**–**c**) Callus of tobacco somatic embryogenesis. (**d–f**) Shooting stage of tobacco somatic embryogenesis. (**g**–**i**) Mature plantlet regenerated from tobacco callus. Red arrow indicates the GUS staining site of *KdSOC1* gene. SAM: shoot apical meristem, CL: cotyledon. HL: hypocotyls.

Interestingly, substantial *KdSOC1* gene expression was detected in the entire early domed-like somatic embryo (similar to globular stage during zygotic embryogenesis) of the tobacco leaf callus (Fig. 1c). However, during the callus shooting stage before the cotyledon extends (similar to the heart stage of zygotic embryogenesis), the GUS protein stained areas were focused in the margin and center of the SAM of the tobacco somatic embryo (Fig. 1f). When the cotyledon and hypocotyl extended, *KdSOC1* gene expression was detected within the SAM area of the newly formed tobacco plantlet (Fig. 1g–i). Taken together, the *KdSOC1* gene was expressed in the globular to heart stages of tobacco somatic embryogenesis and was mainly localized to the SAM.

**Figure 2:**
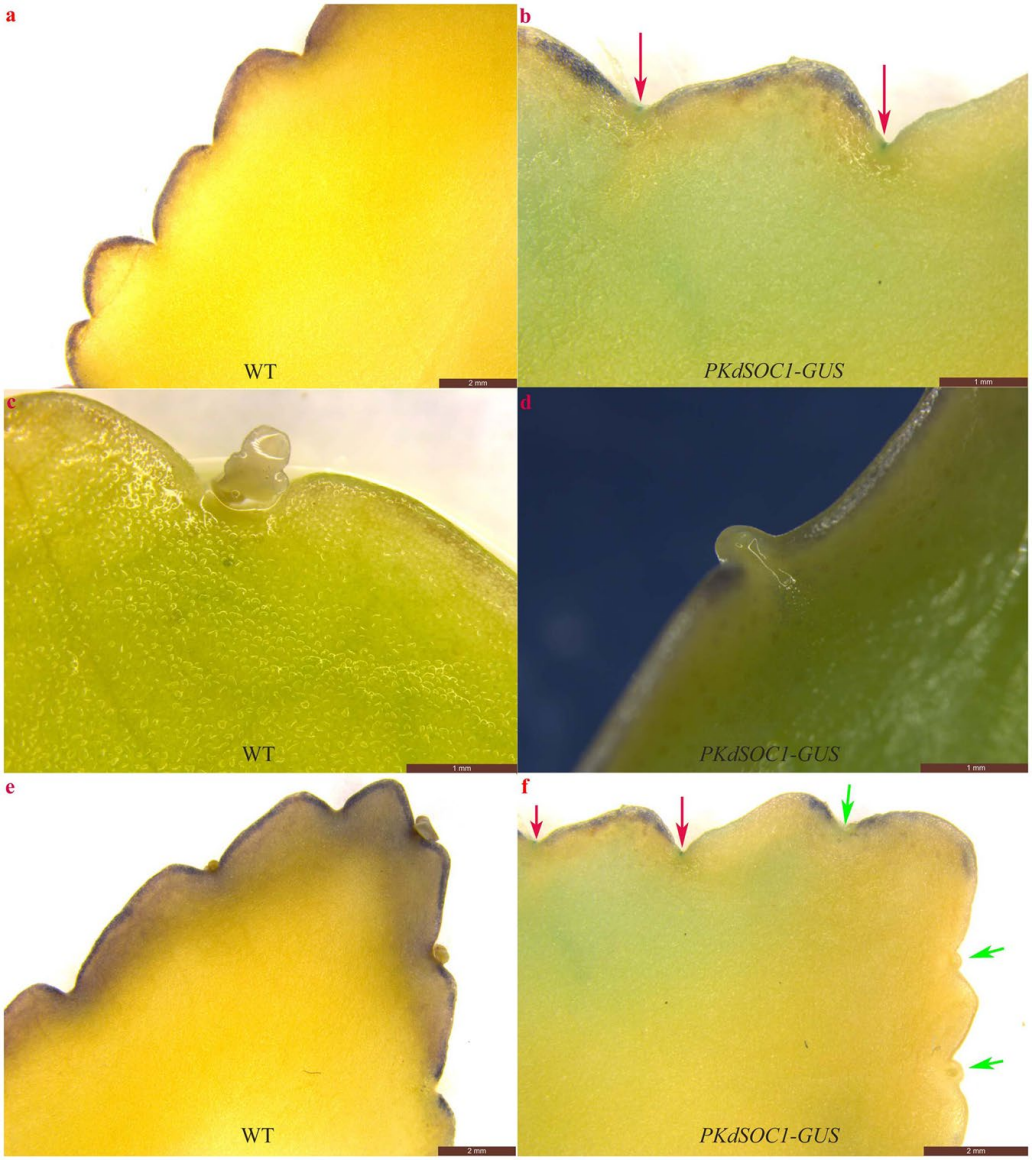
*KdSOC1* gene expression profiles during *Kalanchoe daigremontiana* plantlet morphogenesis. (**a,b**) 14 days old leaves which only formed leaf serration. (**c,d**) 28 days old leaves which formed pedestal sites between each two leaf serration. (**e,f**) 37 days old leaves which formed plantlets along leaf margin.

The *KdSOC1* gene expression profiles during *Kalanchoe daigremontiana* plantlet morphogenesis were also analyzed with the same GUS protein staining method. *KdSOC1* gene expression was detected at the center between two leaf serrations when leaf serrations began to form along the leaf margin of *Kalanchoe daigremontiana* (Fig. 2b). After the pedestal site (which forms from the two leaf serrations and harbors the “stem cell niche” to initiate plantlet formation) appeared with the torpedo-like stage somatic cell, *KdSOC1* gene expression vanished from the entire pedestal site region (Fig. 2d). Because of the unequal growth rate of plantlets, which formed sequentially from the tip side to the bottom side of the leaf, *KdSOC1* gene expression was only detected between leaf serration where no pedestal site formed (Fig. 2f, red arrow).

Taken together, the *KdSOC1* gene was expressed within the SAM area before the late heart stage of tobacco callus or before the formation of the *Kalanchoe daigremontiana* pedestal site, but expression decreased sharply after the cotyledon expanded in tobacco shoot formation and *Kalanchoe daigremontiana* plantlet formation. Thus, this gene may have an role during the early development stage of somatic embryogenesis.

### Disrupting auxin distribution arrests *KdSOC1* gene expression during tobacco callus and shoot induction

**Figure 3:**
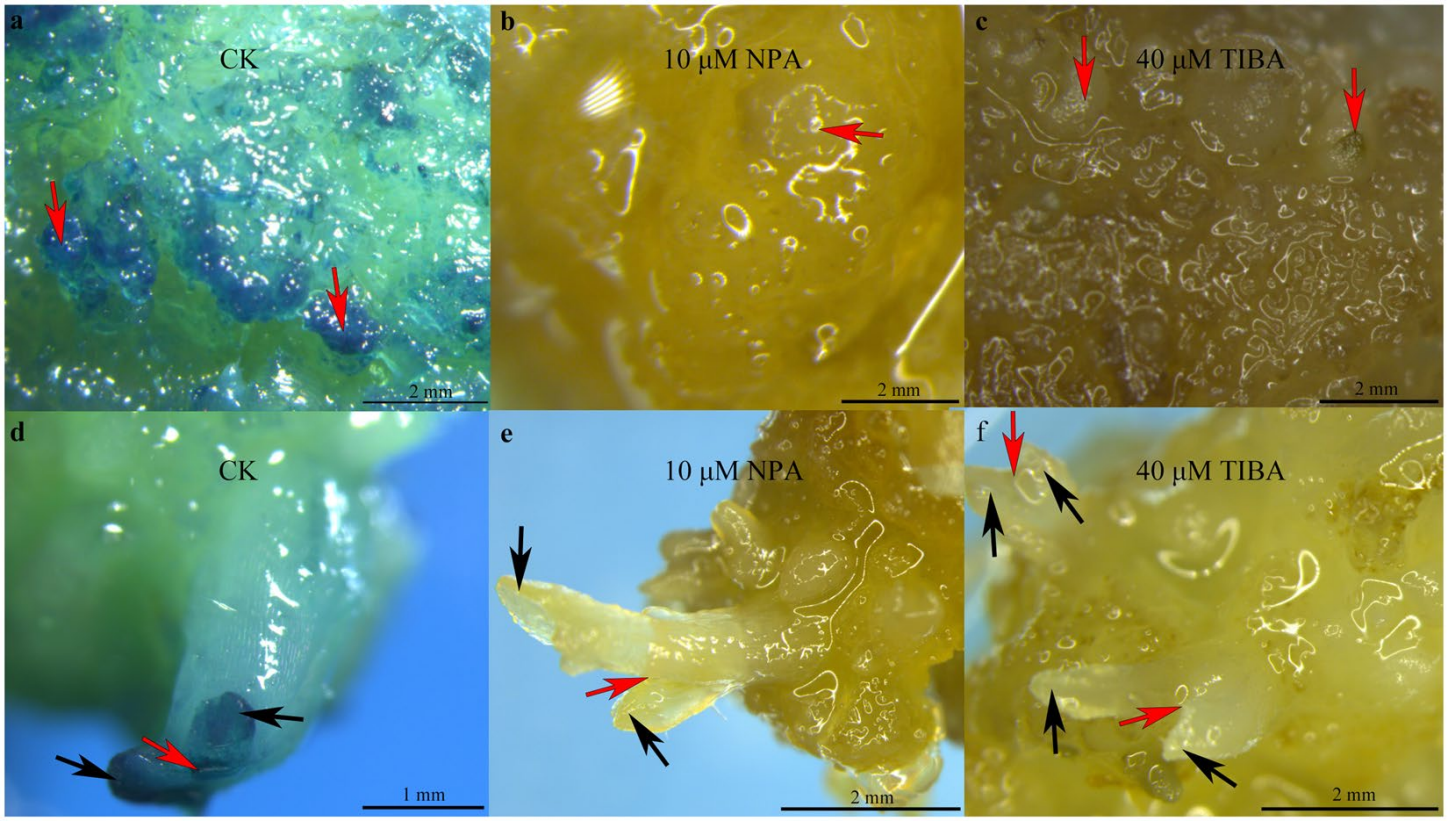
TIBA and NPA disrupted the spatial expression of *KdSOC1* gene promoter driving *GUS* reporter gene during tobacco somatic embryogenesis. (**a**) Normal callus induction medium (CK) without TIBA or NPA. (**b**) Callus induction medium with 10 μM NPA. (**c**) Callus induction medium with 40 μM TIBA. (**d**) Normal shoot induction medium (CK) without TIBA or NPA. (**e**) Shoot induction medium with 10 μM NPA. (f) Callus induction medium with 40 μM TIBA.

According to the well-studied auxin signal pathway model during embryogenesis, we wondered whether disrupting auxin distribution could affect the spatial expression of the *KdSOC1* gene during somatic embryogenesis. Thus, N-1-naphthylphthalamic acid (NPA) and 2,3,5-triidobenzoid acid (TIBA), which are auxin transport inhibitors, were used in tobacco callus and shoot induction media to test whether the *KdSOC1* gene promoter driving the *GUS* reporter gene would remain activated (Fig. 3).

During the tobacco somatic embryo domed-like stage, 10 μM NPA (Fig. 3b) and 40 μM TIBA (Fig. 3c) totally suppressed GUS staining around the somatic cell compared to that under the normal (CK) condition (Fig. 3a), whereas 10 μM NPA (Fig. 3e) and 40 μM TIBA (Fig. 3f) repressed the GUS staining area around the SAM-like area during the tobacco callus shooting stage (near the somatic embryo torpedo stage), compared to those under the CK condition (Fig. 3d).

In general, disrupting the auxin distribution during tobacco somatic embryogenesis interrupted spatial expression of the *KdSOC1* gene, suggesting that the proper auxin concentration between cells is a precondition for synthesizing the KdSOC1 transcript.

### *KdSOC1* gene knockdown results in failed regeneration of transgenic plants

After the *KdSOC1* gene knockdown (RNAi) line transformation was accomplished, most calluses regenerated from infected leaves arrested in the globular stage, even on shoot-inducing medium (Figure S1b,d) compared to that during the control (used as empty vector) regeneration procedure (Figure S1a,c). Few calluses initiated cotyledons, but the cotyledons dried out (Figure S1d) and failed to maintain further organ development, which resulted in the absence of the *KdSOC1* gene RNAi lines of *Kalanchoe daigremontiana*.

Thus, the *KdSOC1* gene was essential for somatic embryogenesis, and knockdown prevented cotyledon maintenance and further organ development.

### Asymmetric plantlet formation and leaf morphological parameters in OE plants

**Figure 4:**
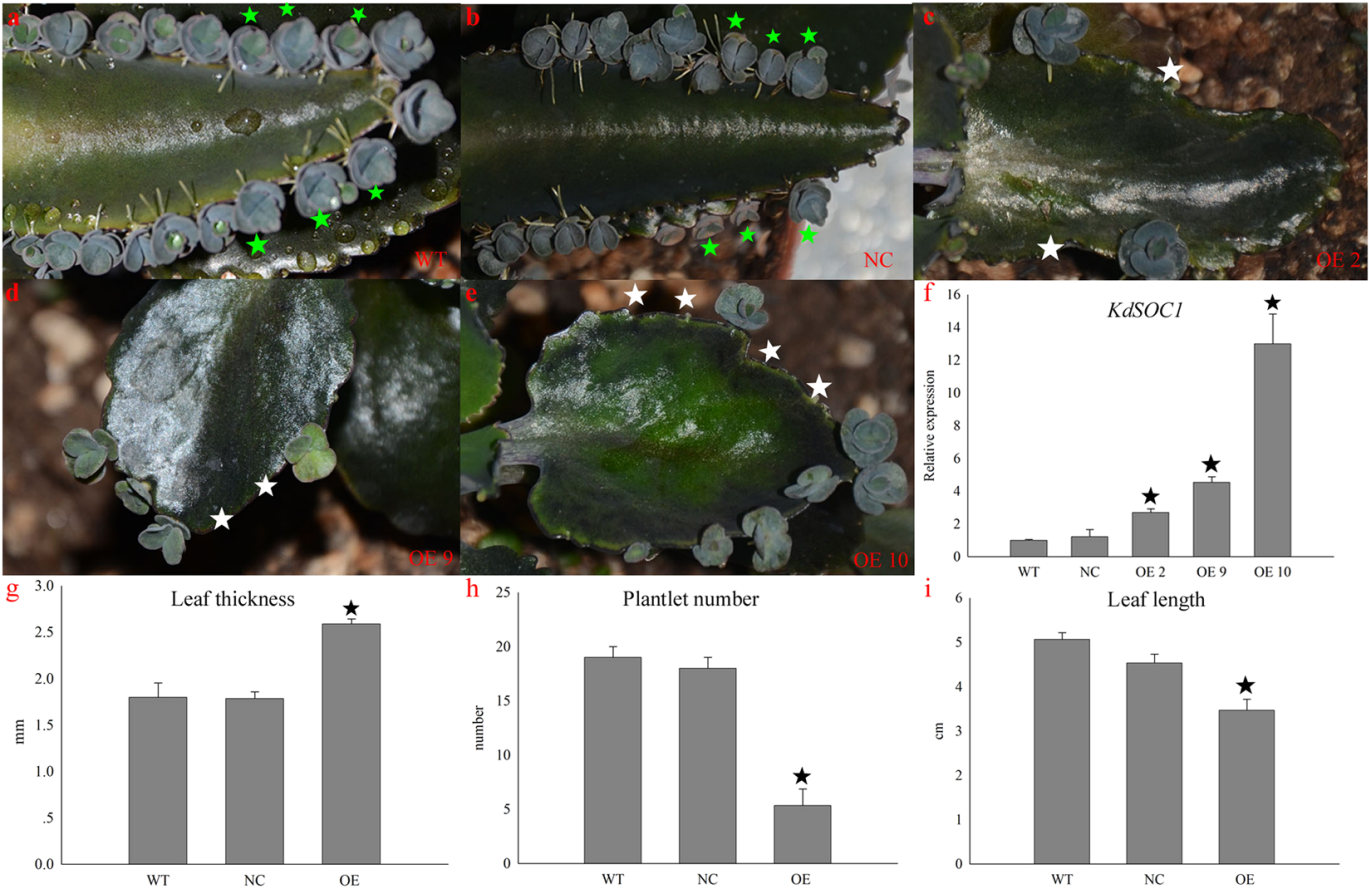
Asymmetric distribution of plantlets along leaf margin in *KdSOC1* OE lines. (**a**) Normal symmetrical plantlets distribute along WT plant leaf margin. (**b**) Normal symmetrical plantlets distribute along NC plant leaf margin. (**c–e**) Asymmetric distribution of plantlets along OE line 2, 9, 10 plant leaf margin. (**f**) *KdSOC1* gene expression in WT, NC and OE plant leaves. (**g–i**) Leaf thickness, plantlet number and leaf length in WT, NC and OE plants. White pentagram indicates the loss of pedestal site for plantlet formation and green pentagram indicates the symmetric plantlet. Dark pentagram indicated significantly different from the WT (*P* < 0.05). Errors bars represent ± SD (standard deviations) of three independent replications.

We speculated that *KdSOC1* might be a key regulator of plantlet formation in *Kalanchoe daigremontiana* based on its expression pattern. We over-expressed the *KdSOC1* gene with the *35 S* promoter to identify the mechanism for modulating plantlet morphogenesis. After screening the positive transformation lines (compared to wild-type [WT] plants) using the *KdSOC1* expression level and quantitative real-time polymerase chain reaction (RT-qPCR), differences in plantlet formation were found along the leaf margins in three OE individual lines (OE lines 2, 9, and 10; OE 2, OE 9, and OE 10) (Fig. 4f).

The WT (Fig. 4a) and negative control (NC: transformed with empty vector; Fig. 4b) plants developed normally shaped leaves and a symmetrical distribution of plantlets formed along the leaf margin with almost equal expression of the *KdSOC1* gene (Fig. 4f). However, a fleshy leaf shape and asymmetrical distribution of plantlets were found along the leaf margin in the lines with *KdSOC1* OE genes 2, 9, and 10 (Fig. 4c–e) (white pentagrams in Fig. 4c–e). *KdSOC1* expression levels in the OE lines were higher than those in WT or NC plants (Fig. 4, right panel); however, the higher expression level of the gene did not positively correlate with the severity of the alternate leaf and symmetrical plantlet distributions (Fig. 4c,e). Mean leaf thickness in OE lines 2, 9, and 10 was much higher than that in WT and NC plants (Fig. 4g). Mean leaf length in OE lines 2, 9, and 10 was much shorter than that in WT and NC plants (Fig. 4i). To our surprise, the mean numbers of plantlets in OE lines 2, 9, and 10 plants were four times lower (p < 0.05) than those of WT and NC plants (Fig. 4h).

**Figure 5:**
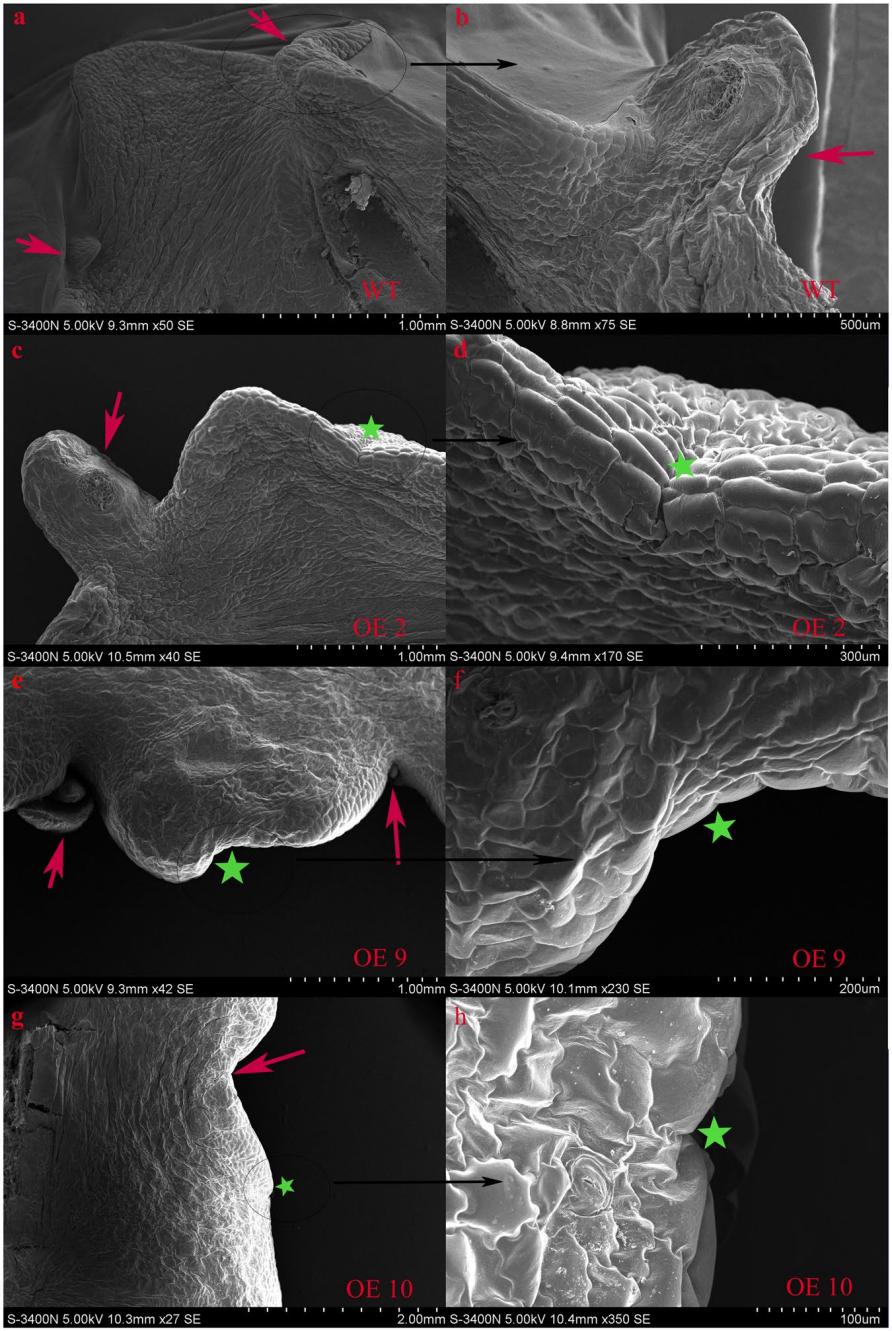
Asymmetric plantlet distribution was formed along the leaf margin of *Kalanchoe daigremontiana* by SEM scanning. (**a,b**) Normal zygomorphous distribution of plantlet was along WT leaf margin. (**c,e,g**) Asymmetric plantlet distribution was formed along *KdSOC1* gene OE plant leave. (**d,f,h**) Plantlet formation pedestal site was disappeared beside leaf margin. Dark pink arrow indicates normal zygomorphous or non-isometric plantlet formation site. Green pentagram indicated absence of pedestal site for plantlet formation.

The defect in symmetrical plantlet distribution in the *KdSOC1* gene OE plants suggested that the pedestal site with the “stem cell niche”, which gives birth to plantlets, might be missing. The symmetrical plantlet formation pedestal sites were detected by scanning electron microscopy along the leaf margins of WT plants (Fig. 5a,b, red arrow). The leaf margins of OE line 2 (Fig. 5c) contained only one pedestal site (dark pink arrow), whereas the other zygomorphous pedestal site (Fig. 5d) was abolished (green pentagram). The same side of the leaf margin in OE line 9 had two non-isometric plantlet formation pedestal sites (Fig. 5e, dark pink arrow) and one missing pedestal site (Fig. 5f, green) between them. The early developing stage of OE line 10 leaf also manifested a zigzag margin (Fig. 5g, dark pink arrow) and a missing zigzag margin (Fig. 5h, green pentagram).

Applying exogenous auxin to the leaf vein stimulates the formation of vascular tissue and influences vascular patterning^21^. We were interested in determining whether OE of the *KdSOC1* gene in the leaves would change the vascular structure.

The thickness of vascular fiber around the leaf serrations in cross-sections of the *KdSOC1* OE line (Figure S2b) and WT leaves (Figure S2a) showed significant difference. The average thickness of vascular fiber near leaf serration in *KdSOC1* OE leaf was higher than that of in WT leaf (Figure S2c).

In summary, *KdSOC1* gene OE resulted in an asymmetric plantlet distribution phenotype because the essential plantlet formation pedestal site was aborted. Besides, the *KdSOC1* gene OE resulted in increased thickness of vascular fiber near leaf serration.

### High auxin content and *PIN1* gene expression in OE plants

*CUC2*, the *KANADI* family genes, and the *PIN1* gene control *Arabidopsis* leaf number, leaf shape, the vascular pattern, and responder organ formation by regulating auxin response factor and auxin response transport activities^22, 23^. In addition, key master genes for maintaining the SAM, such as *WUSHEL* (*WUS*), and *SHOOTMERISTEMLESS* (*STM*), are also essential for controlling leaf shape patterns^24, 25^. Therefore, we checked the indole-3-acetic acid (IAA) and zeatin contents as well as *KdPIN1*, *KdCUC1*, *KdSTM*, and *KdWUS* gene expression profiles in WT, NC, and OE plant leaves.

**Figure 6:**
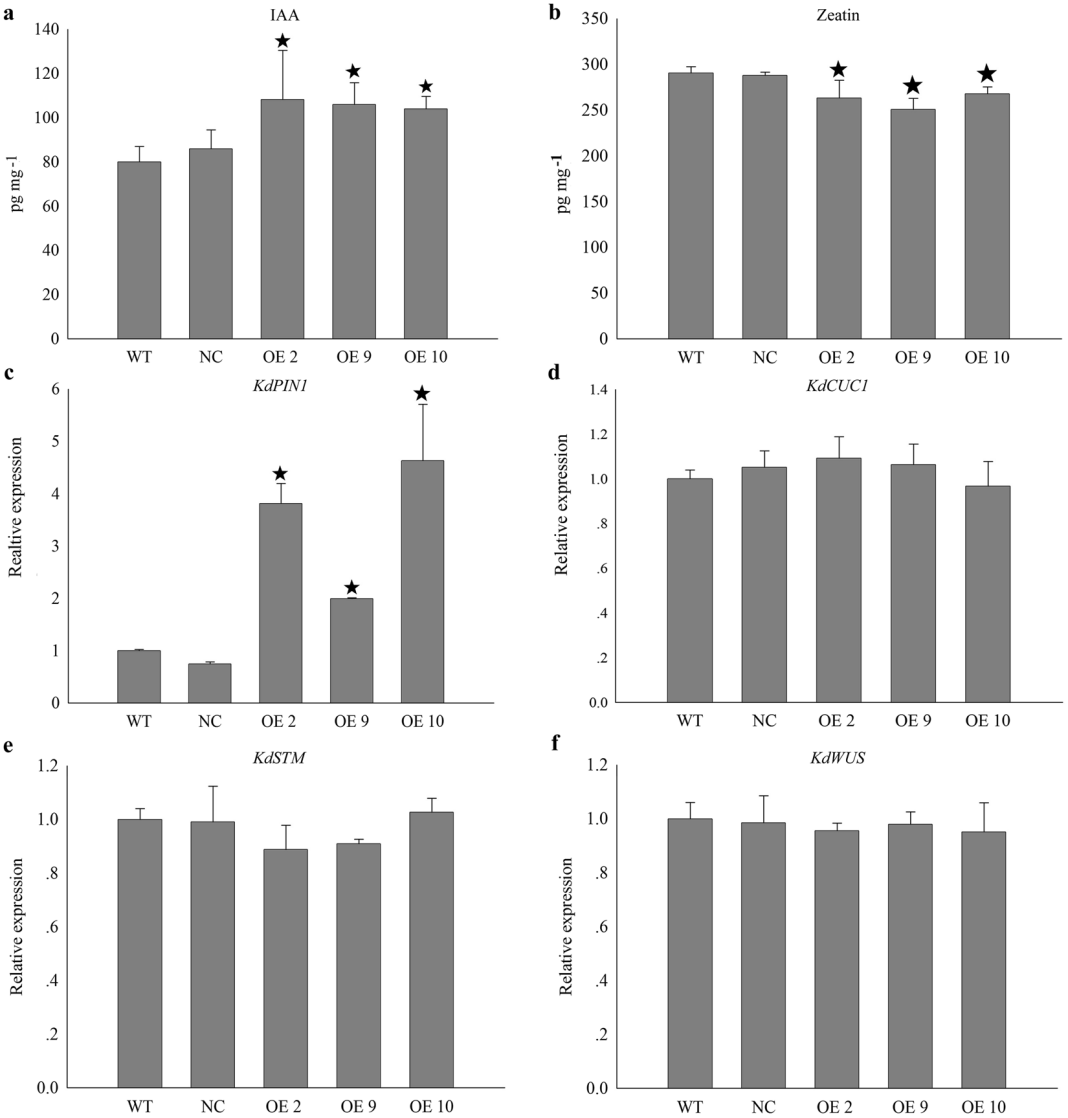
Hormone concentration and embryogenesis related gene expression profiles in leaves of four-month-old *Kalanchoe daigremontiana* OE lines. Dark pentagram indicated significantly different from the WT (*P* < 0.05). Errors bars represent ± SD (standard deviations) of three independent replications.

IAA concentrations produced by OE lines 2, 9, and 10 were slightly higher than those of the WT and NC plants (Fig. 6a). However, zeatin concentrations in OE lines 2, 9, and 10 were slightly lower than those of WT and NC plants (Fig. 6b). Higher *KdPIN1* gene expression levels were detected in the OE lines than in WT and NC plants, which was consistent with the higher IAA concentrations in the OE lines, which may have re-arranged the auxin response along the leaf margin. Surprisingly, expression levels of the *KdCUC1*, *KdSTM*, and *KdWUS* genes, which are well-known key regulators for maintaining SAM identity and the SAM lateral organ boundary, were not different in WT, NC, and OE plant leaves (Fig. 6d–f).

In addition, *WUS*, *STM*, *PIN1*, *AS1*, and *KNOTTED 1* (*Kn1*) were also analyzed during *KdSOC1* gene OE and WT tobacco callus embryogenesis. The *WUS* and *STM* genes, which maintain the meristem identity of somatic cells during the globular stage of embryogenesis in the callus, were expressed at significantly lower levels in the *KdSOC1* gene OE callus at the globular stage compared to that in the WT callus (Figure S3a,b). The *AS1* gene, which is a lateral organ initiation regulator, exhibited no significant difference (Figure S3c) in expression level at this stage, but expression of the auxin response efflux controller *PIN1* decreased tremendously (Figure S3d). The *WUS*, *STM*, *AS1*, and *PIN1* genes all showed high level expression levels in *KdSOC1* gene OE callus (Figure S3c) during the callus late heart stage, compared to those in the WT callus, whereas *Kn1* was not expressed (Figure S3d).

Taken together, higher IAA and lower zeatin concentrations, including higher *KdPIN1* gene expression levels in the OE lines might explain the missing pedestal site to initiate plantlet formation and the asymmetric distribution of plantlets by breaking the hormone balance. *KdSOC1* gene does have the potential ability to affect the auxin response through different ways.

### The *KdSOC1* gene changes the auxin response pattern during somatic embryogenesis of *Arabidopsis*

Considering the asymmetric distribution of plantlets and the relatively high auxin concentration in the *KdSOC1* gene OE lines, we hypothesized that the normal auxin response pattern required for somatic embryogenesis might be dysfunctional. Therefore, OE of the *KdSOC1* gene was generated in *Arabidopsis DR5-GUS* reporting lines to observe the auxin response pattern between *35S-KdSOC1-DR5-GUS* and *DR5-GUS* callus genotypes (WT was used as control).

**Figure 7:**
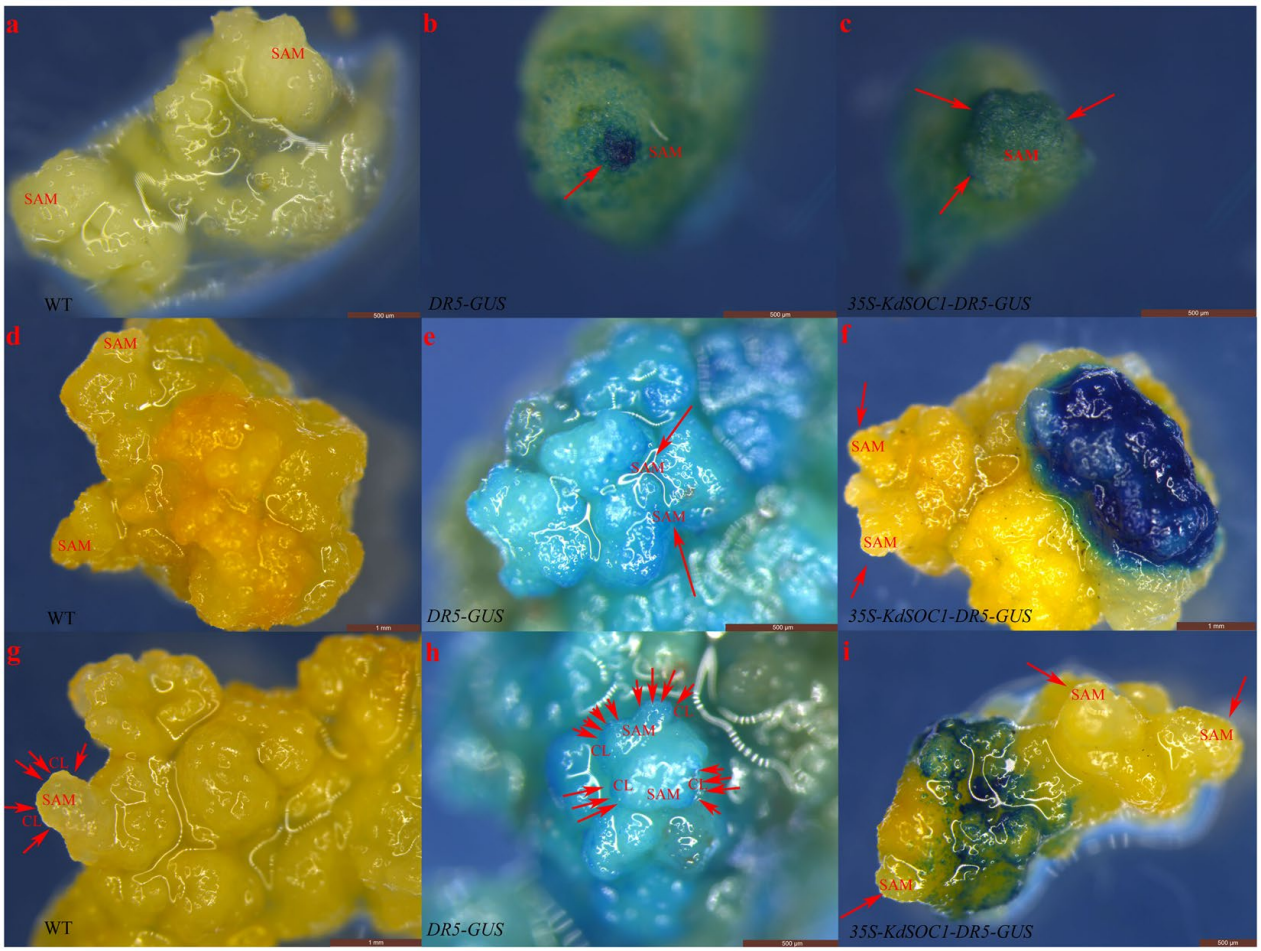
Over-expression of *KdSOC1* gene disturbed the auxin flow during *Arabidopsis* embryogenesis. (**a**–**c**) Early globular stage of *Arabidopsis* somatic embryogenesis. (**d–f**) Early heart stage of *Arabidopsis* somatic embryogenesis. (**g–i**) Torpedo stage of *Arabidopsis* somatic embryogenesis. Red arrow indicated the high auxin concentration site and white arrow indicated the missing auxin concentration site. SAM: shoot apical meristem, CL: cotyledon.

The auxin response during the early stage of *DR5-GUS* callus formation (Fig. 7b, red arrow) was concentrated in the SAM-like tissue, whereas the auxin response in the *35S-KdSOC1-DR5-GUS* callus was dispersed in a much wider area (Fig. 7c, red arrow) around the SAM-like structure. The auxin response at a later stage was concentrated more between two unstretched cotyledons in the *DR5-GUS* callus (Fig. 7e, red arrow), whereas the auxin response in the *35S-KdSOC1-DR5-GUS* callus (Fig. 7f) was biased within a single area. The auxin response in the *DR5-GUS* callus (Fig. 7h, red arrow) was typically distributed along the cotyledon margin at the early shooting stage of somatic embryogenesis, but the auxin response of the *35S-KdSOC1-DR5-GUS* callus was similar to the last stage. The growth phase changed more slowly in the *35S-KdSOC1-DR5-GUS* callus than in the *DR5-GUS* callus (data not shown), which may have been the result of the *KdSOC1* gene OE disturbance.

In summary, *KdSOC1* gene OE changed the auxin response pattern during *Arabidopsis* somatic embryogenesis, which was essential for initiating organ formation and the change in growth phase during somatic embryogenesis.

## Discussion

Our results suggest that plantlets formation accompanied with the leaf serration and pedestal site appearance might be possible with proper auxin response. The leaf shape in *Arabidopsis* is regulated by *PIN1* (auxin efflux transporter) and *CUC2* (stimulated by *PIN1*-dependent auxin flow and inhibited by auxin)^26^. According to this classical model, plantlet morphogenesis process in *Kalanchoe daigremontiana* also reflects similar characteristics (the SAM structure harbored in the pedestal site between two leaf serrations appeared and plantlet initiated). Thus, we deduced that a *KdSOC1*-auxin module orchestrated plantlet formation in *Kalanchoe daigremontiana*.

**Figure 8:**
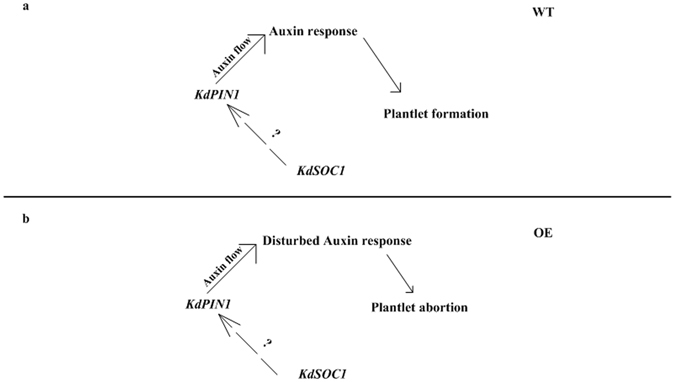
Putative model of *KdSCOC1* gene mediated plantlet morphogenesis. (**a**) Plantlet morphogenesis in WT plant. (**b**) Plantlet morphogenesis in *KdSOC1* gene OE plant. Green arrow indicated auxin flow and green represented the maximum auxin concentration.

The plantlet formation process was possibly completed by the *KdSOC1* gene-mediated auxin response pattern through the *PIN1* gene (Fig. 8a). The *KdSOC1* gene up-regulated the *KdPIN1* gene expression through a unknown way (Fig. 8a). Then, *KdPIN1* gene might function as a transporter to control auxin flow direction which was essential for shaping leaf serration that harbored the pedestal site and initiated plantlet. The certain auxin flow distribution at leaf pedestal site caused certain auxin response which was crucial for the development of stem niche within pedestal site and further leaf development. The *KdSOC1* gene OE in the leaf might disturb the auxin efflux through the excessive *KdPIN1* gene expression (Fig. 8b). The change in auxin efflux possibly resulted in certain auxin response which was unfavorable for initiating the stem niche. Therefore, OE plant leaves had no stem niche pedestal site, resulting in the plantlet formation defective phenotype. Thus, *KdSOC1* gene function during plantlet formation process is possibly to tune the auxin response. Future work on *KdSOC1* gene network is another step further to study plantlet formation.

According to previous study in observing endogenous hormones concentrations during plantlet formation in *Kalanchoe daigremontiana*, increasing accumulation of both auxin and cytokinin at the asexual reproduction site (pedestal site) indicated their positive roles in plantlet development^16^. In addition, this study also found *KdCYCB1* gene (plays essential role in regulating cell G2/M and G1/S phase transition^27^) up-regulation during plantlet initiation, which suggested that the auxin and cytokinin accumulation in pedestal site might reactivate the cell cycle^16^. While, other extensive studies also showed that auxin signaling was positive for cell division^28^. Thus, increasing *KdSOC1* gene expression might coincide with the auxin signaling during early plantlet formation in *Kalanchoe daigremontiana*.

Another gene function study in *Arabidopsis axr2-1* [INDOLE-3-ACETIC ACID 7 (IAA7)/ AUXIN RESISTANT 2 (AXR2), one auxin signaling component that regulated flowering time in *Arabidopsis*] mutant also suggested reduction of free auxin might act to inhibit the timing of floral transition under short days by negatively regulating the expressions of the *SOC1* and other related genes^29^. Therefore, *SOC1* gene expression might respond to proper auxin signaling, which was essential for the organ development in right timing. In this study, the *KdSOC1* gene was mainly expressed within the SAM until the cotyledon extends or the pedestal site appears, suggesting that *KdSOC1* gene assistance was only needed during the globular to heart stage of somatic embryogenesis in the SAM of the callus or before the pedestal site was initiated. The *AtSOC1* gene expression pattern detected previously by *in situ* hybridization shows that it first appears in the SAM and leaf primordia and then is only detected in the floral meristem but not the inflorescence meristem^30^. This *AtSOC1* gene expression pattern was elucidated by its multiple cis-acting element in the promoter^31, 32^. Therefore, the *KdSOC1* and *AtSOC1* gene expression patterns suggest that initiating vegetative or sexual organ formation might both be affected by auxin signal transduction. The downstream auxin responsive genes are key, as they control different developmental events during plant growth.

The *KdSOC1* gene might affect SAM differentiation like the *AGAMOUS* (AG) gene (MADS domain protein) function in *Arabidopsis* floral stem cell fate. A mutation in the *AtAG* gene results in a defect in floral stem cell fate termination and the floral organ identity specification, which identifies the AG protein together with Polycomb group proteins repress *WUS* expression by methylating histone H_3_ Lys-27 of *WUS* ^33^. We also detected repressed *WUS* and *STM* gene expression during the globular stage of somatic embryogenesis in the *KdSOC1* gene OE tobacco callus (Figure S3), suggesting that the *KdSOC1* gene terminates the stem cell fate and promotes leaf initiation for a subsequent growth stage during somatic embryogenesis. Therefore, a potential *KdSOC1* gene function of stimulating a change in the growth phase during somatic embryogenesis may occur by manipulating the auxin response, which could possibly change cytokinin/auxin ratio in the SAM.

A mutation in the *AtSOC1* gene caused bolting arrest and late flowering in *Arabidopsis* compared to those in WT plants^30^. However, knockdown of the *KdSOC1* gene severely disturbed organ initiation after somatic embryogenesis. Such different phenotypes resulting from a homologous gene mutation suggest that *Kalanchoe daigremontiana* possesses a distinct regulation module for plantlet morphogenesis. The lack of function of the *KdLEC1* gene demonstrates that not all essential growth-related genes are required in all species^15^. Although genetic information might be inherited from the same ancestor, the continuous adaptation of *Kalanchoe daigremontiana* in tropical areas may have conferred the *KdSOC1* gene with the novel function to affect plantlet formation^34^. Therefore, further research is needed to discover the *KdSOC1* gene network and related hormone signaling to understand the detailed molecular mechanism of plantlet morphogenesis.

## Conclusion

This study focused on *KdSOC1* gene function during asexual leaf plantlet morphogenesis in *Kalanchoe daigremontiana*. *KdSOC1* gene expression was detected for the first time during *in vitro* somatic embryogenesis and plantlet morphogenesis. Knockdown of the *KdSOC1* gene resulted in a defect in cotyledon formation during the early heart stage of somatic embryogenesis. OE of the *KdSOC1* gene resulted in asymmetric plantlet distribution. *KdSOC1* gene OE in *Arabidopsis DR5-GUS* reporting lines resulted in different auxin response patterns during different stages of somatic embryogenesis. In summary, the *KdSOC1* gene was determined to regulate the auxin responsive pattern and affected *Kalanchoe daigremontiana* plantlet formation. Such gene function might be crucial for the plasticity of plantlet formation under various environmental conditions.

## Experimental procedures

### Plant material and growth conditions

Tobacco (*Nicotiana tabacum*) and *Kalanchoe daigremontiana* were grown in substrate (peat:perlite = 3:1) under a 16/8-h light (250 μmol m^−2^ s^−1^) cycle at 25 °C at 50–70% relative humidity in a growth room. *Arabidopsis thaliana* plants were grown under 16/8-h light (250 μmol m^−2^ s^−1^) cycle at 20 °C at 75–80% relative humidity in a growth room.

### Vector construction and transformation

*KdSOC1* gene complete opening reading frame (ORF) cDNA sequence was introduced into pBIN438^35^ plasmid driven by enhanced CaMV35s promoter as over-expression (OE) construct; RNAi construct of *KdSOC1* gene was designed by introducing antisense and sense ORF of *KdSOC1* gene into pZH01^36^ plasmid with partial *GUSA* gene as intron between them; tissue expression construct of *KdSOC1* gene (*PKdSOC1-GUS*) was designed by introducing its 1.2 kb promoter sequence to replace CaMV35s promoter in pBI121^37^ plasmid and drives the expression of *GUS* gene (empty pBI121 plasmid [*35S-GUS*] was used as positive control). Then all the plasmids mentioned above were all transformed in *Agrobacterium tumefeciens* strain LBA4404.

Tobacco transformation (*PKdSOC1-GUS* and *35S-GUS* constructs were used) procedure was undergoing with previously described method^38^. The selection of tobacco positive transformant after LBA4404 infection was divided into shoot induction (with 50 mg ml^−1^ Kanamycin for selection pressure) and root induction (with 100 mg ml^−1^ Kanamycin for selection pressure) processes^38^. *Kalanchoe daigremontiana* transformation (OE, RNAi and *PKdSOC1-GUS* constructs were used) method was followed by previously described by Garces^39^. The selection of *Kalanchoe daigremontiana* positive transformant after LBA4404 infection was divided into shoot induction (with 50 mg ml^−1^ Kanamycin for selection pressure) and root induction (with 100 mg ml^−1^ Kanamycin for selection pressure) processes^39^. *Arabidopsis* plant (harboring *DR5-GUS* reporting genes) transformation (OE construct was used) using the *Agrobacterium*-mediated floral dip method^40^.

### Confirmation of positive transgenic plant

The confirmation of positive transformation in tobacco T1 generation of each construct was performed by PCR amplification of the *KdSOC1* gene promoter. The confirmation of positive transformation in *Kalanchoe daigremontiana* was performed by RT-qPCR amplification of the *KdSOC1* gene ORF. The confirmation of positive OE transformation in *DR5-GUS Arabidopsis* T1 generation was performed by PCR amplification of the *KdSOC1* gene ORF.

### Tissue culture of transgenic tobacco and *Arabidopsis*

The top leaves from T1 generation OE and P*KdSOC1*-*GUS* positive transformants were surfaced sterilized^39^ and cut into 0.5 cm diameter leaf discs. Each leaf disc was first subjected on callus induction Murashige and Skoog (MS) medium with 1 mg ml^−1^ 6-BA, 0.5 mg ml^−1^ NAA and 50 mg ml^−1^ Kanamycin for 2 weeks (for auxin distribution repression assay, 40 μM TIBA and 10 μM NPA were added respectively). The newly formed callus was then transferred to MS medium with 1 mg ml^−1^ 6-BA, 0.1 mg ml^−1^ NAA and 50 mg ml^−1^ Kanamycin for 3 weeks until the shoot appeared (for auxin distribution repression assay, 40 μM TIBA and 10 μM NPA were added respectively).

The immature seed of *Arabidopsis* transgenic plants (*DR5-GUS*; *35S-KdSOC1-DR5-GUS*; WT) were used as explants in somatic tissue culture follow the previously described method^41^.

### Microscopy and photography

Scanning electron microscopy for *Kalanchoe daigremontiana* was performed as previously described^42^. Phenotype of transgenic plant pictures were captured by Canon digital camera (Japan) The photos of different inducing stage of tobacco callus were taken by Leica MDG41 microsystem (Singapore). All photographs were adjusted assembled into figures using Adobe Photoshop 4.0 (AdobeSystems, San Jose, CA, USA). The vascular fiber thickness was measured based on ImageJ software.

### GUS staining assay

Histochemical localization of GUS activity was performed as previously described^43^. Images are representative of > 10 observed samples stained in three independent experiments.

### RT-qPCR analysis of transgenic plants

RNA extractions were performed on three biological replicates of T1 generation transgenic tobaccos expanding leaves using the TRIzol^®^ (Invitrogen, USA), while RNA extractions for *Kalanchoe daigremontiana* were performed^44^. All samples were treated using RQ DNAse (Promega, USA) and were ethanol precipitated. cDNA synthesis was performed using GoScript^TM^ Reverse Transcription Systemfor RT-PCR (Promega, USA) with 2 µg RNA and using the oligo (dT) primer. RT-qPCR was performed using the TransStart® Tip Green qPCR SuperMix (TransGenBiotech, China), following the manufacturer instructions and an ABI Step One PCR instrument (Applied Biosystems, USA) was used. 200 ng cDNA was used for each reaction. The results were calculated using the 2^−∆∆CT^ method^45^. The *KdActin* were used as housekeeping gene and the primers used in RT-qPCR were listed in Table S1.

### Hormone detection in transgenic plants

Raw hormone mixtures were extracted from 0.1 g of tissue from the uppermost fully developed leaf with 1 mL of pH 7.0 PBS. Indole-3-acetic acid (IAA) and cytokinin concentrations were measured using a colorimetric ELISA kit containing plates pre-coated with antibody specific to IAA or cytokinin. (Winter Song Boye Biotechnology Co. Ltd., Beijing). Samples were diluted six-fold prior to the tests. The standard curve was generated based on a series of known IAA or zeatin standard sample reactions to 180, 120, 60, 30, 15, and 0 ng mL^−1^. Three individual lines from each transgenic plant and WT were used.

### Statistical analyses

ANOVA and mean comparisons were performed using SPSS version 20.0 software. The value of 2^−ΔΔCT^ was also proceeded with SPSS version 20.0 software. Error bars represent standard deviation. Black stars letters indicate statistically significant differences between OE and WT at *P* < 0.05 based on Duncan’s multiple range test.

### Data availability

The authors declare to provide all the data when requested.

## Electronic supplementary material

Supplementary information.
