# Supplementary material for: Over-expression of KdSOC1 gene affected plantlet morphogenesis in Kalanchoe daigremontiana
Source: Sci Rep. 2017 Jul 17;7:5629. doi: 10.1038/s41598-017-04387-0 (PMC5514138; doi:10.1038/s41598-017-04387-0)
Supplement: Supplementary file 1 — Supplementary information. [file 41598_2017_4387_MOESM1_ESM.doc]

**Over-expression of *KdSOC1* gene affected plantlet morphogenesis in *Kalanchoe daigremontiana***

**Chen Zhu^1#^, Li Wang^2#^, Jinhua Chen^3^, Chenglan Liu^3^, Huiming Zeng^*3^, Huafang Wang^*1^**

**^1^** National Engineering Laboratory for Tree Breeding, College of Biological Sciences and Technology, Beijing Forestry University, Beijing, China. **^2^** Sivilculture Forestry department, College of Forestry, Beijing Forestry University, Beijing, China. **^3^** Turfgrass Management department, College of Forestry, Beijing forestry university, Beijing, China.

**^#^ These authors contributed equally to this article.**

**Corresponding authors E-mail:** sciinfo@bjfu.edu.cn**;**

hfwang@bjfu.edu.cn

**Supplement Figure**

**
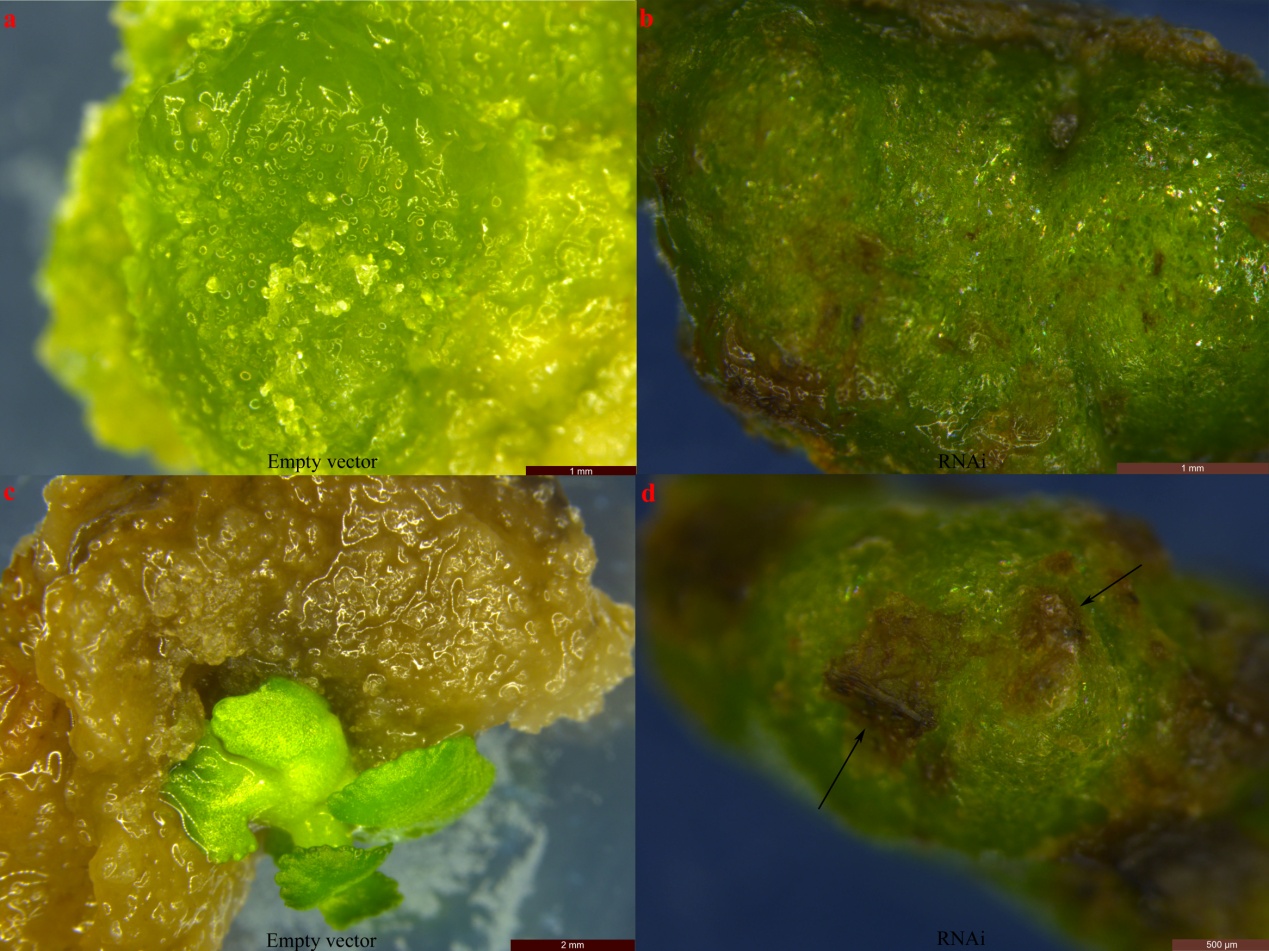
**

**Figure S1** *KdSOC1* gene knock down resulted in failed regeneration of transgenic *K. daigremontiana* plant. (a) Callus induction stage of empty vector infected leaf explants. (b) Callus induction stage of *KdSOC1* gene RNAi vector infected leaf explants. (c) Shoot induction stage of empty vector infected leaf explants. (d) Shoot induction stage of RNAi vector infected leaf explants. Black arrow indicated dry cotyledon.

**
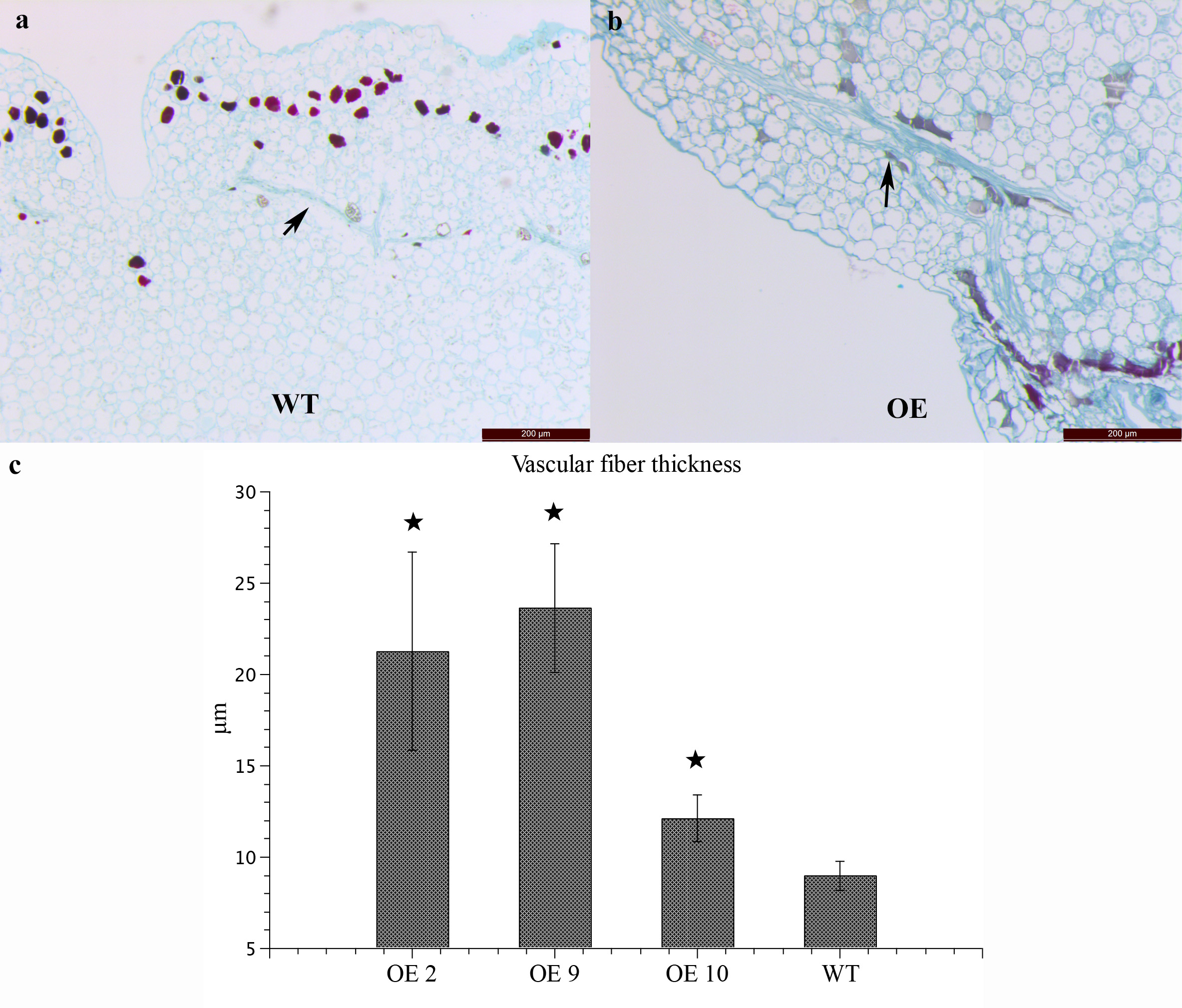
**

**Figure S2** *KdSOC1* gene OE promoted vascular fiber thickness near leaf serration in *Kalanchoe daigrematiana*. (a) Upper leaf epidermal structure of WT. (b) Upper leaf epidermal structure of *KdSOC1* gene OE. (c) Vascular fiber thickness of WT and *KdSOC1* gene OE. Black arrow indicated the vascular fiber near leaf serration. Errors bars represent ± SD (standard deviations) of three independent replications. Black stars indicated significantly difference between OE and WT (*P* < 0.05).

**
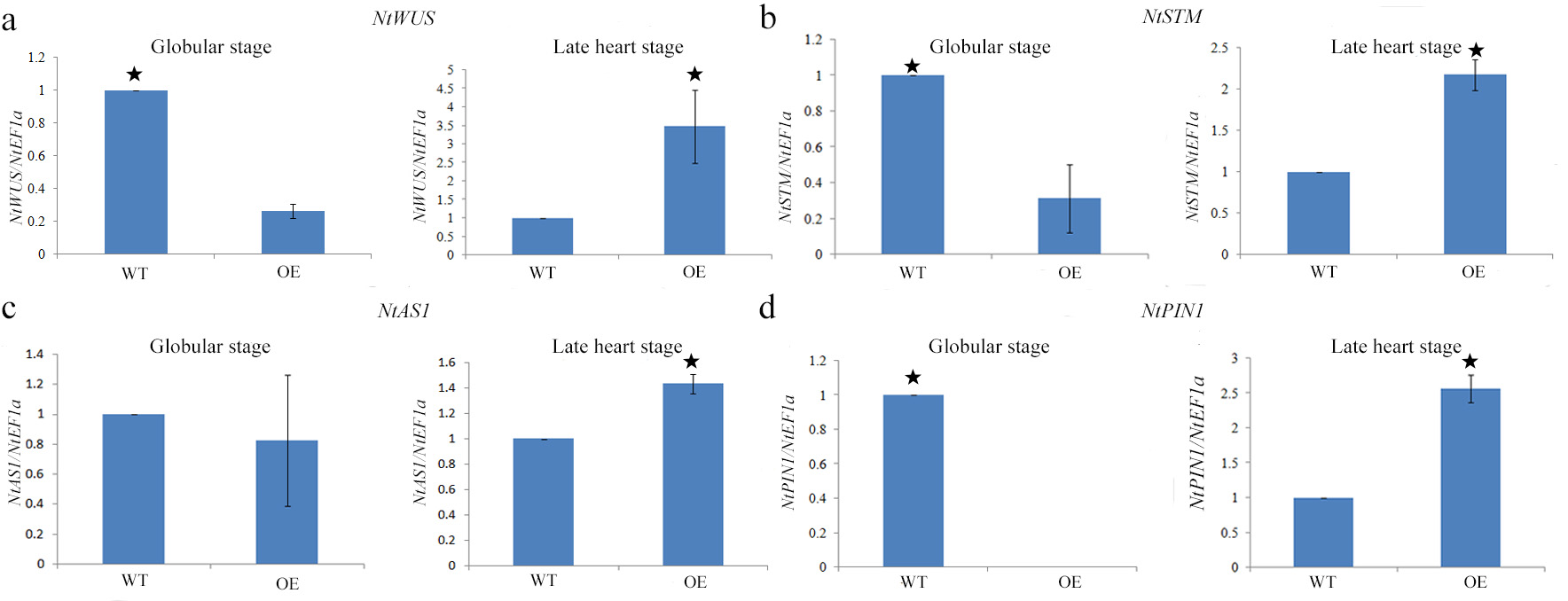
**

**Figure S3** Embryogenesis related genes expression during different growing stages of plantlet induction from tobacco callus. Errors bars represent ± SD (standard deviations) of three independent replications. Black stars indicated significantly difference between OE and WT (*P* < 0.05).

| **Primer ued in plasmid construction** |  |
| --- | --- |
| *KdSOC1* gene OE expression plasmid | F (*BamH*I): 5' CGGGATCCATGGTGCGAGGGAAGACTCAG 3' |
|  | R (*Sal*I): 5' GCGTCGACGTTGCTTCCATTGTTGGAGCA 3' |
| Tissue expression plasmid (*PKdSOC1-GUS* ) | F (*Hind*III): 5' CCAAGCTTGTCGAGTGAAATGAACAT 3' |
|  | R (*Xba*I): 5' GCTCTAGATCTCTCTACCTGCTCCCTCT 3' |
| *KdSOC1* gene RNAi expression plasmid: anti sense | F (*Sal*I): 5' :GCGTCGACATGGTGCGAGGGAAGACTCAG 3' |
|  | R (*BamH*I): 5' CGGGATCCGTTGCTTCCATTGTTGGAGCA 3' |
| *KdSOC1* gene RNAi expression plasmi: sense | F(*Bgl*II): 5' GAAGATCTATGGTGCGAGGGAAGACTCAG 3' |
|  | R (*Spe*I): 5' GGACTAGTGTTGCTTCCATTGTTGGAGCA 3' |
| **Primer used in RT-qPCR** |  |
| *NtAS1* (Gene Bank ID: JQ686933.1) | F: 5'GAAGTTGTGAAAGAGCGGAGTG 3' |
|  | R: 5' AGAAGAGTTTGTGGCGAAGGA 3' |
| *NtSTM* (Gene Bank ID: JQ686930.1) | F: 5' TATCCATCGGAATCCCAGAA 3' |
|  | R: 5' GAGCAGCATCCATTACCACAA3' |
| *NtPIN1* (Gene Bank ID: HM102422.1) | F: 5' AAAGTTGAAGGGCAAAGAAAC 3' |
|  | R: 5' GTCATTACACTTGTTGGAGGC 3' |
| *NtKn1* (Gene Bank ID: AF544052.1) | F: 5' CAACAACCACCACCTCTTTCA 3' |
|  | R: 5' GGGTATTCCACCAATCCAGCA 3' |
| *NtWUS* (Gene Bank ID: JQ686923.1) | F: 5' TTGATGAAACCCTAGTAGACGA 3' |
|  | R: 5' CTGCCAATGAAAGAGTTGAGAC 3' |
| NtEF1a (Gene Bank ID: D63396.1) | F: 5' CCTCTTGACCCGCAGTTACAT 3' |
|  | R: 5' TGATTGGTGCAGATCCCTCTA 3' |
| *KdSTM* (Gene Bank ID: DQ674268.1) | F: 5' TCCTCACTACCACCGTCTCGT 3' |
|  | R: 5' GCATCTCACAGTAAGCCTCCAT 3' |
| *KdActin*（Gene Bank ID: GQ339777.1） | F: 5’ GACTATGAGGCTGAGTTGGAGAC 3’ |
|  | R: 5’ TCAATGAAGGCTGGAAAAGG 3’ |
| *KdWUS* （Guo, J., Liu, H., He, Y., Cui, X., Du X and Zhu, J. Origination of asexual plantlets in three species of Crassulaceae. Protoplasma 252, 591-603 (2015).) | F: 5' ATCAACCAGCAGGTAACAGTCAT 3' |
|  | R: 5' TAGCTCAGCTTTTCAAAACAACA 4' |
| *KdCUC1*（Guo, J., Liu, H., He, Y., Cui, X., Du X and Zhu, J. Origination of asexual plantlets in three species of Crassulaceae. Protoplasma 252, 591-603 (2015).) | F: 5' GATAAGTCCGCTTTGAAGAGCAGGG 3' |
|  | R: 5' GTTTTCTTCATTCCCACTACTACCA 3' |
| *KdSOC1* (In submission) | F: 5' TCTGTGATGCTGAGGTTGCTCT 3' |
|  | R: 5' CCTTCGACTGGCTATCTTTCGT 3' |
| *KdPIN1* (In submission) | F: 5' GCTATGACCATGATTACGCCA 3' |
|  | R: 5' GCGAAAACAAAGGGAACAATT 3' |

**Supplementary table S1** Primer used in this assay.
